# Detection of Metastatic Disease in Cardiophrenic Lymph Nodes: FDG PET/CT Versus Contrast-Enhanced CT and Implications for Staging and Treatment of Disease

**DOI:** 10.3389/fonc.2013.00260

**Published:** 2013-10-01

**Authors:** Shannon Farmakis, Kaveh Vejdani, Razi Muzaffar, Nadeem Parkar, Medhat M. Osman

**Affiliations:** ^1^Department of Radiology, Saint Louis University, Saint Louis, MO, USA; ^2^Division of Nuclear Medicine, Department of Radiology, Saint Louis University, Saint Louis, MO, USA; ^3^Saint Louis VA Medical Center, Saint Louis, MO, USA

**Keywords:** F18-FDG, PET/CT, cardiophrenic, lymph node metastasis, TNM staging

## Abstract

**Objective**: To determine whether FDG PET/CT was more sensitive than CT in detecting metastatic disease in the cardiophrenic space and whether the presence of disease in this location would change the staging and clinical management.

**Materials and Methods**: About 1200 PET/CT scans were retrospectively reviewed over 20 months for the presence of FDG-avid cardiophrenic lymph nodes. The SUVmax was used to quantify the metabolic activity in each of the lymph nodes. The radiographic data was used for correlation. A retrospective review of diagnostic CT reports performed within a 1-month period of time of the PET/CT in the same subset of patients determined whether cardiophrenic lymph nodes were mentioned.

**Results**: About 9 (0.8%) of the 1200 studies were found to have FDG-avid cardiophrenic lymph nodes (four males and five females with a mean age of 55 years; range 7–69, median 59). The mean SUVmax was 2.4 (range 1.2–7.9; median 1.9). Only three of the patients were found to have suspicious lymph nodes on CT. The presence of cardiophrenic lymph nodes had the potential to change the staging and/or management in three of the patients.

**Conclusion**: PET/CT is more accurate in the detection of pathologic cardiophrenic lymph nodes than CT, especially when they are subcentimeter in size. When present, staging and/or management was potentially affected in 33%. Therefore, these nodes should be included in the TNM staging classification.

## Introduction

The presence or absence of disease within lymph nodes has important prognostic significance in patients with known malignancy as it can alter the stage of their disease and even change management from surgical cure to non-surgical management. The numerous locations of lymph nodes and their various implications in disease staging has been well-documented in the TNM classification system for many types of cancers, and while abnormal mediastinal lymph nodes play a critical part in the staging of diseases, such as lung cancer, lymphoma, ovarian cancer, or other extrathoracic primary malignancies, very little has been done to determine the importance of cardiophrenic lymph nodes in disease staging. In fact, there is very little data in the literature on what constitutes a normal sized cardiophrenic lymph node. Also, while metastatic disease involvement of the cardiophrenic lymph nodes is a well-documented phenomenon in ovarian cancer, these nodes are not included in the lymph node maps of the most recent IASLC for lung cancer staging (1).

Computed tomography (CT) is routinely used in the evaluation of and treatment planning for patients with malignancy as well as for surveying patients for recurrent disease. 18-FDG Positron Emission Tomography/Computed Tomography (PET/CT) is also used for these purposes and is predominantly used to determine the extent of disease involvement and evaluate for the presence of distant nodal metastases. The cardiophrenic angle is an area that is often overlooked on routine CTs, especially since there is such variability in their presence and size. PET/CT, on the other hand, is able to detect abnormal FDG activity in a very small node without being limited by size criteria.

The purpose of this study was to determine whether PET/CT is more sensitive than CT in detecting metastatic disease in the cardiophrenic space and whether the presence of disease in this location would change the staging and clinical management of patient with malignancy.

## Materials and Methods

### Patient selection

A retrospective review of 1200 consecutive FDG PET/CT reports of cancer patients in the span of 20 months was performed to detect the presence of FDG-avid cardiophrenic lymph nodes. The maximum standardized uptake value (SUVmax) was used to quantify the metabolic activity in each of the lymph nodes. The radiographic data was used for correlation. A retrospective review of diagnostic contrast-enhanced CT reports performed within a 1-month period of time of the PET/CT in the same subset of patients determined whether the cardiophrenic lymph nodes were mentioned. Only one of the CTs was performed without contrast.

### PET/CT scanning

An intravenous 5.18 MBq/kg (0.14 mCi/kg) injection of ^18^F-FDG was administered after the patient had fasted at least 4 h. Patients sat in a quiet room without talking during the subsequent 60 min of the FDG uptake phase. Blood glucose level was <200 mg/dl in all patients. All scans were acquired using a PET/CT scanner (Gemini TF; Philips Medical Systems) with an axial co-scan range of 193 cm.

### CT scanning

The CT component of the PET/CT scanner consisted of a 64 slice multidetector helical CT with a gantry port of 70 cm. Parameters were as follows for 20–21 bed acquisitions: 120–140 kV and 33–100 mAs (based on body mass index), 0.5 s per CT rotation, pitch of 0.9, and 512 × 512 matrix. CT data were used for image fusion and the generation of the CT transmission map. In all patients, the arms were placed above the patient’s head for CT acquisition except in patients with head and neck cancers where the arms were placed at the patient’s sides. The CT images were obtained without oral or IV contrast.

### PET scanning and image processing

The PET component of the PET/CT scanner is composed of Lutetium-Yttrium Oxyorthosilicate (LYSO)-based crystals. Emission data were acquired on average for 20–21 bed positions (193 cm coverage, identical to the CT protocol). Emission scans were acquired at 1–2 min per bed position. The FOV was from the top-of-head to the bottom-of-feet in all patients. The 3-dimentional whole body (WB) acquisition parameters consisted of a 128 × 128 matrix and an 18 cm FOV with a 50% overlap. Processing consisted of the 3D Row Action Maximum Likelihood Algorithm (RAMLA) method (2). Total scan time per patient was 20–45 min.

### Image analysis

PET/CT images of the nine patients included were retrospectively evaluated on the Gemini TF extended brilliance workstation (EBW) by two board certified nuclear medicine physicians. A log recorded reports which documented the presence of cardiophrenic lymph nodes based on PET and unenhanced CT. When seen, the size and SUVmax of the lymph nodes were obtained. Cardiophrenic lymph nodes were defined as those lymph nodes located anterior to the pericardium and within 2 cm of the diaphragm. These have alternatively been described as anterior prepericardiac nodes and are responsible for draining the diaphragm, pleura, anterior abdominal wall, and liver through the internal mammary chain (3). No minimum size, number, or standardized uptake value criteria was assigned to the lymph nodes for inclusion into the study. Recent CT reports were then reviewed to determine whether the cardiophrenic lymph nodes were seen and mentioned in the CT report. A thorough review of the medical record including all clinical notes and radiology reports was performed on each patient to determine the type of malignancy and any planned or completed methods of treatment.

## Results

Of the 1200 patients evaluated, 9 (0.8%) were found to have FDG-avid cardiophrenic lymph nodes (four males and five females with a mean age of 55 years; range 7–69; median 59). On the CT portion of the PET/CT the nodes demonstrated a mean size of 0.8 cm in the short axis diameter (range 0.4–1.6 cm; median 0.7 cm). The mean SUVmax was 2.5 (range 1.2–7.9; median 1.9). Only three of these patients were found to have suspicious lymph nodes on CT (two contrast-enhanced CT, one non-contrast CT). The presence of cardiophrenic lymph nodes did not affect the staging and management in six out of the nine patients as they already had widely metastatic disease, but their presence did have the potential to affect staging in the remaining three patients. The table summarizes the disease processes and lymph node characteristics (Table 1).

**Table 1:** **Summary of patient and lymph node characteristics**.

Age (years)	Sex	Type of cancer	Size of cardiophrenic lymph nodes (cm)	SUVmax of cardiophrenic lymph nodes
56	M	Lung squamous cell carcinoma; also history of HIV and granulomatous disease	0.4	1.5
52	F	Pancreatic adenocarcinoma	0.4, 0.4	2.3, 1.6
59	M	Glioblastoma multiforme	1.5, 1.6, 1.6	1.6, 0.7, 0.6
63	M	Large B-cell lymphoma	0.8	1.6
56	F	Lung squamous cell carcinoma	0.9, 0.6, 0.5	3.0, 1.2, 2.0
7	F	T cell lymphoma	0.8	3.0
68	F	Pancreatic adenocarcinoma	0.7, 0.6	3.9, 2.9
72	F	Large B-cell lymphoma	0.4, 0.5, 0.8, 0.5	1.8, 2.3, 7.9, 1.4
63	M	Pancreatic adenocarcinoma	1.1	3.8

**Figure 1:**
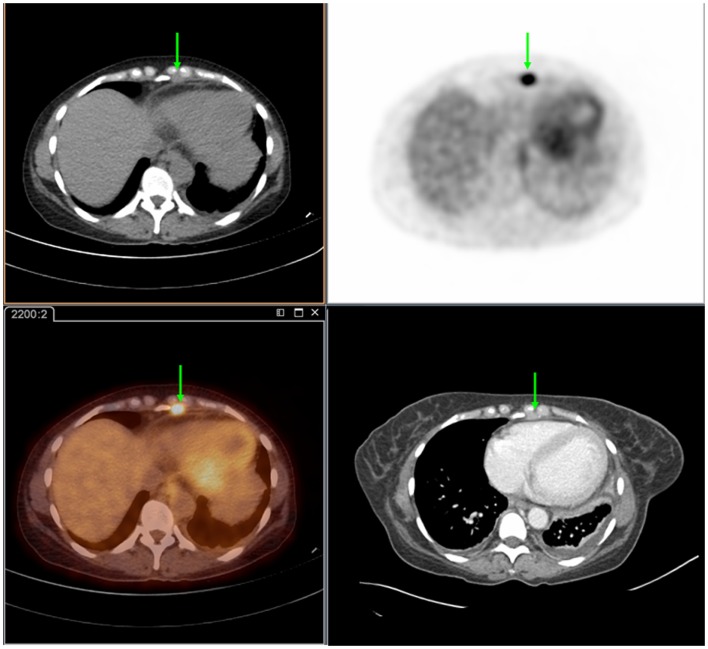
**Seventy-two year-old female presenting with metastatic pancreatic adenocarcinoma**. PET/CT demonstrates an FDG-avid cardiophrenic lymph node on non-contrast CT (top left), PET (top right), and fused images (bottom left). These were not commented on in the contrast-enhanced CT (bottom right). Differences in the levels between the two studies are due to patient positioning and respiratory motion.

The size of the lymph nodes did not have an impact on the suspicion of disease involvement as many of the lymph nodes were <0.5 cm (39%). However, the likelihood of disease in the lymph nodes was thought to directly correlate to the SUVmax value (Figure 1). The presence of more than one cardiophrenic lymph node was also considered to be more indicative of disease involvement.

**Figure 2:**
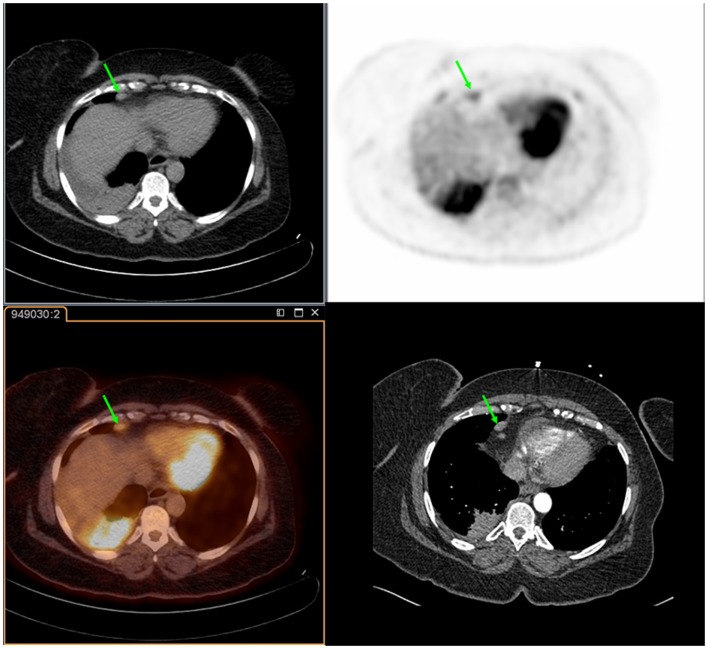
**Fifty-six year-old female with a history of squamous cell carcinoma of the right lung**. PET/CT demonstrates an FDG-avid cardiophrenic lymph node on non-contrast CT (top left), PET (top right), and fused images (bottom left). These were not commented on in the contrast-enhanced CT (bottom right). Differences in the levels between the two studies are due to patient positioning and respiratory motion.

None of the patients underwent biopsy of the cardiophrenic lymph nodes. As a result, pathologic correlation was not available. However, most of the patients had biopsy-proven malignancies from other sites. These included squamous cell carcinoma of the lung, pancreatic adenocarcinoma, large B-cell lymphoma, and T cell lymphoma. One of the patients did undergo biopsy of several lung lesions which were pathologically proven to be necrotizing granulomas. This patient had a complex history including HIV and previously treated cavitary squamous cell carcinoma of the lung, and the presence of FDG-avid cardiophrenic lymph nodes may have been a result of granulomatous disease or recurrent cancer. Subsequent studies on this patient over the course of 22 months demonstrated no change in size of the lymph node with a gradual decrease in activity thereby making an inflammatory process more likely. An additional patient had a primary diagnosis of glioblastoma multiforme (GBM) of the brain with additional findings of splenomegaly, multiple enlarged abdominal lymph nodes, and several FDG-avid cardiophrenic lymph nodes. This patient died before workup of a possible diagnosis of lymphoma was able to be performed. In this patient, the presence of supradiaphragmatic FDG-avid lymph nodes could have affected staging if an additional primary malignancy was able to be diagnosed. A third patient was found to have biopsy-proven squamous cell carcinoma of the right lung with ipsilateral mediastinal and subcarinal lymph node involvement. In addition, the patient had ipsilateral FDG-avid cardiophrenic lymph node involvement (Figure 2). The TNM classification for lung cancer does not include cardiophrenic lymph nodes in its staging map. It is unclear whether the presence of this abnormal lymph node had the potential to change the staging in this patient.

## Discussion

Most solid lesions detected within the cardiophrenic space represent lymph nodes. More often than not, the presence of these lymph nodes is abnormal and should be evaluated further for malignancy from primary tumors (supra- or infra-diaphragmatic) (4). Usually, the underlying etiology is lymphoma since there is a large lymphatic drainage system located along the diaphragm. Alternatively, since this region is also responsible for abdominal lymphatic drainage, numerous abdominal neoplasms can metastasize to the cardiophrenic region. In addition, primary lung or pleural malignancies can spread via the supradiaphragmatic lymph nodes. Since dissemination of tumor into lymph nodes usually implies metastatic disease, their presence commonly affects the treatment of the patient (4). Furthermore, involvement of these lymph nodes by lymphoma has been shown to be infrequent; therefore, their presence is important to document as one study found it altered treatment (5, 6).

Also, while small lymph nodes in this region may be a normal finding, there is very little data on what size actually constitutes an abnormal lymph node. Aronberg et al. proposed a cutoff of 5 mm for a normal cardiophrenic lymph node. He noticed that nodes >6 mm were seen in patients with diseases associated with lymphadenopathy (7). Sussman et al. on the other hand, suggested a size of 1 cm as the upper limit of normal unless more than one cardiophrenic lymph node measuring <1 cm was present which would then be considered suspicious (3). Subsequently, Dorfman et al. reviewed previous studies evaluating the size of paracardiac lymph nodes defined as those within 2 cm of the diaphragm and adjacent to the pericardium and found a range for normal lymph nodes of 2–8 mm in the short axis diameter (2). His conclusion was that lymph nodes that exceeded a short axis diameter of 8 mm in the paracardiac region were abnormal and should be considered suspicious, especially if more than one node was present (2). If a size >0.8 cm had been used as a cutoff for suspecting disease within a lymph node in this study, multiple abnormal lymph nodes would have been missed. In fact, many of the abnormal lymph nodes were <0.8 cm (72%). Furthermore, all but three of these lymph nodes were missed on the CT studies. The three that were reported on the CTs measured 1 cm, 1.4 cm, and 0.5 cm suggesting that most were identified simply because of their larger size. The additional use of FDG PET allowed for the detection of abnormal activity within the lymph nodes thereby increasing the likelihood of disease involvement. The presence of more than one lymph node, regardless of size, was also more likely to imply pathology. The degree of SUV uptake appeared independent of the lymph node size; however, the degree of uptake did appear to correlate with the likelihood of pathology in the lymph node. For example, multiple subcentimeter cardiophrenic lymph nodes were seen in a patient with known large B-cell lymphoma who was being evaluated after therapy. Four lymph nodes (size 0.4–0.8 cm) demonstrated variable activity (SUVmax 1.4–7.9) suspicious for disease progression.

This study finds that PET/CT is much better than contrast-enhanced CT at detecting pathology in smaller cardiophrenic lymph nodes. It also finds that size alone is not adequate for ruling out pathology in lymph nodes in the cardiophrenic region as lymph nodes as small as 0.4 cm can harbor disease.

The presence of abnormal cardiophrenic lymph nodes had the potential to change staging in three of the nine patients in this study. The most significant potential was in a patient with squamous cell carcinoma of the right lung (maximum diameter 7.5 cm) who also had ipsilateral mediastinal and subcarinal lymph node involvement. Normally, this patient would have been classified as Stage IIIA by the TNM staging system and would, therefore, be able to potentially undergo resection (8). However, the presence of disease in several cardiophrenic lymph nodes has the potential to alter the staging to a Stage IIIB or Stage IV, thus rendering the treatment as non-operable. Unfortunately, the IASLC lung cancer staging project does not include cardiophrenic lymph nodes in the nodal staging map. Potential classifications would be to characterize the nodal staging based on whether the cardiophrenic nodes are ipsilateral or contralateral, and, if contralateral, should they be classified as a N3 lesion or as a M1 lesion.

Another problem in determining whether a cardiophrenic lymph node harbors disease is that it is a location that is not easily biopsied. Oftentimes, this deters physicians from trying to sample tissue from these nodes. However, in the situation in which this is the only abnormal lymph node, biopsy becomes a necessity for treatment planning. Several reports document the findings of abnormal cardiophrenic lymph nodes representing metastases, either isolated or multiple, from various gynecologic malignancies, most commonly epithelial ovarian carcinoma (9–11). The presence of these lymph nodes had variable prognostic significance depending largely on the presence of additional disease involvement, such as peritoneal metastasis or other abnormal extra-abdominal lymph nodes. The most commonly used method to biopsy these lymph nodes in the past has been video-assisted thoracic surgery (VATS). For patients undergoing cytoreductive surgeries for ovarian cancer, this meant an additional surgery. Recently, a newer method has been described which is performed at the same time as the cytoreductive surgery. A small incision is made in the diaphragm in order to biopsy the abnormal cardiophrenic nodes (transabdominal cardiophrenic lymph node dissection or CPLND) (12). This method may potentially be performed in other abdominal malignancies in which the cardiophrenic lymph node may represent a change in staging and a surgery is already being performed in the abdomen, such as in lymphoma, hepatocellular carcinoma, pancreatic adenocarcinoma, etc. (13). Furthermore, if large enough, these lymph nodes could potentially be biopsied safely using ultrasound guidance.

This study does have some limitations. First, the study only selected studies in which the words “cardiophrenic lymph node” were mentioned in the report. As a result, there may have been some studies that were missed if the nodes were referred to by another name, such as paracardiac, supradiaphragmatic, or pericardial. Similarly, any FDG-avid cardiophrenic lymph nodes not detected in the reading of a PET/CT would also not be included, and there is no way to estimate the number of these possible cases. The study is also limited by its small sample size. In addition, none of the cardiophrenic lymph nodes in the patients studied had biopsies of the nodes performed to confirm the presence of disease. Also, of the patients died before workup of a possible lymphoma could have been performed. Therefore, no pathologic process was proven in this patient other than the primary brain malignancy (GBM). Furthermore, another patient had a history of both lung cancer and granulomatous disease. The abnormal cardiophrenic lymph nodes in this patient could have been a result of either disease process. The findings of increased SUVmax in a lymph node while indicating an underlying pathology, is not specific for neoplasm versus inflammation. Therefore, the findings of abnormal activity in the lymph nodes were more sensitive when there was a known primary malignancy. Lastly, this study has limitations inherent in those that are retrospective. Ideally, the PET/CT and contrast-enhanced CT scans would have been performed in the same day in order to determine which imaging modality is more effective at detection of the lymph nodes.

## Conclusion

This study finds that PET/CT is more accurate in the detection of pathologic cardiophrenic lymph nodes than CT, particularly when they are smaller than 1 cm. The size of the lymph node was not found to be important as an exclusion criteria for pathology while the presence of multiple lymph nodes in this region was considered more suspicious for disease. In addition, the degree of activity as measured by the SUVmax was thought to directly correlate with the presence of disease. While the presence of abnormal cardiophrenic lymph nodes did not impact staging or management in six of the patients, it did have the potential to impact management in three of them, most notably in the patient with squamous cell carcinoma of the lung. We propose that the IASLC lung cancer TNM staging classification be revised to include the presence of cardiophrenic lymph nodes in the nodal map. Also, while biopsies of these nodes can prove challenging given the location, there may be alternatives to the traditional VATS, such as transabdominal CPLND or ultrasound guided biopsy.

## Conflict of Interest Statement

The authors declare that the research was conducted in the absence of any commercial or financial relationships that could be construed as a potential conflict of interest.
